# On the possible role of protein vibrations in information processing in the brain: three Russian dolls

**DOI:** 10.3389/fnmol.2015.00038

**Published:** 2015-07-23

**Authors:** John Smythies

**Affiliations:** Laboratory for Integrative Neuroscience, Center for Brain and Cognition, University of California, San DiegoLa Jolla, CA, USA

**Keywords:** heteroreceptor complexes, protein vibrations, protein dynamics, colored noise, perturbation waves, allosteric binding, vibration codes, hierarchical computing

## Abstract

Until recently it was held that the neurocomputations conducted by the brain involved only whole neurons as the operating units. This may however represent only a part of the mechanism. This theoretical and academic position article reviews the considerable evidence that allosteric interactions between proteins (as extensively described by Fuxe et al., 2014), and in particular protein vibrations in neurons, form small scale codes that are involved as parts of the complex information processing systems of the brain. The argument is then developed to suggest that the protein allosteric and vibration codes (that operate at the molecular level) are nested within a medium scale coding system whose computational units are organelles (such as microtubules). This medium scale code is nested in turn inside a large scale coding system, whose computational units are individual neurons. The hypothesis suggests that these three levels interact vertically in both directions thus materially increasing the computational capacity of the brain. The whole hierarchy is thus similar to three nested Russian dolls. This theoretical development may be of use in the design of experiments to test it.

## Introduction

Fuxe et al. (2014) have recently proposed that information essential to the laying down of permanent memories in the brain is carried by allosteric waves between the various protein molecules (receptors, ion channels and scaffolding proteins) that make up the post-synaptic density and presynaptic regions (hypothesis A). They propose that binding of a neurotransmitter to its receptor induces a conformational change in the receptor molecule that is transmitted by an allosteric wave to induce conformational changes in the other protein molecules that make up the heteroreceptor complex. This pattern is learned through the resulting reorganization of the various homo- and hetero-receptor protein complexes in the post-perisynaptic membrane into the formation of novel “bar-codes” represented anatomically by the novel heteroreceptor complexes formed, and physiologically by their new balance with the corresponding homoreceptor complexes. In this way a molecular engram for short-term memory is created. In this mechanism, learning involves the sequential modification of the potential for allosteric interactions of down-stream heteroreceptor complexes following novel synaptic inputs that is converted into the groundwork for permanent memories by a process that involves new protein synthesis. For further comprehensive information on protein allosterics and bar-codes see Fuxe et al. (2014) and Smythies (2015).

The purpose of the current article is to explore in detail the possible role of another aspect of protein function—thermal vibrations—during information processing by neurons in addition to the allosteric mechanism. Although protein vibrations are important for intercellular signaling for all cells (Hawkins and McLeish, 2006; Iakhiaev and Iakhiaev, 2013), for the purposes of this review I will concentrate on their role in neurons.

## A Protein Vibration Code

The stable folding pattern of a protein molecule is based on the molecule finding its own lowest free energy. The binding of one protein molecule to another depends on finding the lowest possible free energy of the pair. This involves allosteric factors that include overall shape, complementary binding of charged groups, hydrogen bonding and lipophilic interactions. If one of these groups functions as part of the receptor for an external agent (e.g., a neurotransmitter), then activation of that receptor will change, not only the conformation of that protein, but of the other proteins bound to it. The information relevant to the formation of memories is held by hypothesis (A) to be carried by the microanatomical spatio-temporal patterns of protein molecules in the post-synaptic density, and in vesicle release sites in the presynaptic terminal as described above. These form a species of bar-code.

An additional factor in this process may be the vibration spectra of the constituent proteins (hypothesis B). All molecules thermally vibrate at particular frequencies and emit two types of noise—high frequency “white” noise by small molecules (such as water and metal ions) and medium frequency “colored” noise by large molecules (such as proteins; Al-Khalili and McFadden, 2014). The bends and twists of the peptide chain of proteins are flexible and cause the chain to emit signals composed of colored noise, but only at specific frequencies. The frequency is determined by the aminoacid sequence and the conformation and movements of the protein molecule. Thus each protein will have a signature dynamic pattern of colored noise in the form of a number of peaks in its noise emission spectrum, in which the number, size and frequency of the peaks will vary. Thus, changing these conditions in one protein in a heteroreceptor complex by some stimulus will lead immediately to a change in the conformation and noise emission spectra of all the proteins in the complex. A mechanical model for this system would be a Morse code machine for people who are both deaf and blind. The receiver for such an information transmission system would be a knob that vibrated at different frequencies and/or for different periods.

In a review of the role of global and local vibrational modes in the dynamic allostery of protein Hawkins and McLeish (2006) state “It is now clear that dynamics plays an important role in protein function. For example, there is growing evidence for a dynamic contribution to allosteric signaling within protein molecules.” Furthermore the electric fields produced in folded proteins influence nearly every aspect of protein function (Suydam et al., 2006; Stafford et al., 2010; Ly et al., 2011; Fafarman et al., 2012; Schkolnik et al., 2012; Ritchie and Webb, 2013; Walker et al., 2013).

Using molecular dynamic studies, Iakhiaev and Iakhiaev (2013) have identified a molecular system in protein molecules that forms and propagates “perturbation waves”. This constitutes a network, which transmits energy and information between different parts of the protein that relies on autonomic coherence resonance in atomic fluctuations. This system can coordinate and integrate structural changes in the protein molecule. The authors state:

“Protein dynamics is essential for its [a protein’s] functional activity and includes several types of motions: atomic vibrations, residue motions (including bond stretching and bond angle bending), motions of secondary structure elements, and correlated motions of multiple residues, including motions of large domains (Yonetani and Laberge, 2008; Teilum et al., 2009). These motions carry the signals related to the intramolecular communication, example of which is allosteric regulation of protein function. In allosteric mechanism, ligand binding at an allosteric site is coupled to a structural and/or dynamic change at a distant regulated site of the protein. This implies that there exist links between distant regions of protein which can be defined in terms of long-range intramolecular communication.”

Recent reports have added some direct experimental support for this hypothesis:

- Using orientation-sensitive terahertz near-field microscopy measurements of chicken egg white lysozyme single crystals Acbas et al. (2014) report, direct observation of such long-range protein vibrational modes.
- Müller-Werkmeister and Bredenbeck (2014) state that, whereas theoretical studies predict highly directional anisotropic vibrational energy transfer (VET) in proteins connecting distant functional sites to mediate allosteric communication, experimental support for this has been lacking. These authors tested this hypothesis by ultrafast vibrational pump-probe spectroscopy and observed major instances of such signals in synthetic polypeptides over long distances in an investigation of the structural dynamics related to phototaxis and gene regulation of a photoactive flavoprotein.
- Brust et al. (2013) observed multiple time scales for the dynamics associated with different vibrations of a protein that suggested an underlying hierarchical relaxation pathway.

There have also been reports that thermal vibratory stimuli can induce stem cell differentiation:

- Tong et al. (2013) reported that high frequency vibratory stimulation of induced differentiation of multipotent human mesenchymal stem cells in culture.
- A similar report in the case of human umbilical stem cells was published by Cho et al. (2012).
- Kim et al. (2012) confirm that human mesenchymal stem cells are mechanosensitive to low-magnitude-high-frequency vibration signals such that they could facilitate the osteogenic process. There is evidence that this reaction depends on Wnt signaling (Hou et al., 2011).

This process could form the basis for a code that translates changing inputs into a changed pattern of specific colored noise vibrations via alterations of the conformation of the protein complex. These patterns of vibration could carry a transmissible vibration code (B) in addition to the allosteric anatomical code (A). One output of this mechanism could be the modulation of new protein synthesis by the system that would consolidate memories. Repeated presentation of the stimuli could lead to repeated activations of a signaling pathway from the perisynaptic area to perisynaptic ribosomes, or to the cell nucleus, where it would stimulate the synthesis of new proteins that would be carried back to the synapse to join the heterosynaptic complexes to form the developing basis of permanent memories.

Vibrational interactions between proteins and functionally attached molecules could also be involved. An example of this is contained in a study of protein-heme interactions in cytochrome *c* (Cyt *c*). The active site of Cyt *c* consists of a heme covalently linked to a pentapeptide segment (Cys-X-X-Cys-His), which provides a link between the heme and the protein surface, where the redox partners of Cyt *c* bind. Galinato et al. (2012) performed nuclear resonance vibrational spectroscopy measurements that provided the basis to propose that heme-protein vibrational dynamic couplings play a role in electron transfer by coupling vibrations of the heme directly to vibrations of the protein at the protein–heme interface. This could allow for the direct transduction of the thermal (vibrational) energy from the protein surface to the heme surface.

In a previous article, Smythies (2015) discussed the possible role of the cytoskeleton in neural information processing. I presented a picture of the large-scale computing network in the brain composed of networks of units (neurons) connected by intercellular electrical, chemical and epicrine (exosome) signaling. Then, I suggested that, inside each unit neuron, there seems to be another computing mini-network composed of individual organelles, mainly microtubules, connected by intracellular chemical and electrical signaling, that may act by fine-tuning the unit neuron. The present article proposes that this pattern repeats at an even smaller scale, and that, inside many organelles, there is another minimus-computing computing network composed of individual protein molecules connected by molecular perturbation waves that may operate by fine-tuning the protein molecule—one inside the other like a group of Russian matyoshka dolls.

This hierarchical computing mechanism may represent a significant increase in the computational capacity of the brain. This possibility is discussed by Satinover (2001). The human brain forms a “… nested hierarchy an entire parallel computer one stage being but a processing element in the next larger one.” In one direction in this hierarchical Hopfield net, activation of a receptor on the surface of the neuron at the highest stage induces a change in the structure and function of the cytoskeleton at the middle stage following Hopfield dynamics. This transmits changes to the inner structure and function of the individual proteins at the lowest stage. The causal chain can also travel in the reverse direction.

Sahu et al. (2013b) report that a single brain-neuron-extracted microtubule is a memory-switching element, whose hysteresis loss is nearly zero. Their study shows how a memory-state forms in the nanowire and how its protein arrangement symmetry is related to the conducting-state written in the device, thus, enabling it to store and process ~500 distinct bits, with 2 pA resolution between 1 nA and 1 pA. The authors state that this random access memory is an analog of flash memory switch used in a computer chip. Using scanning tunneling microscope imaging, they further demonstrate how single proteins behave inside the nanowire when this 3.5 billion years old nanowire processes memory-bits. The same group, Sahu et al. (2013a) report that structured water molecules inside the tubulin helix promote the electrical conductivity of the tubulin molecule.

Brinks et al. (2014) have reviewed the use of picosecond and femtosecond laser spectroscopy in a variety of electronic and vibrational dynamic systems e.g., individual fluorophores at room temperature, that showed electronic (de)coherence, vibrational wavepacket interference and quantum control. The authors also present the results of two color phase shaping applied to photosynthetic light-harvesting complexes, which allows investigation of the persistent coherence in photosynthetic complexes under physiological conditions at the level of individual complexes. Further methodological descriptions of UV Raman spectroscopy and localized near-infrared spectroscopy are presented by Tamura et al. (1997) and Balakrishnan et al. (2008) respectively.

## Experiments to Test the Hypothesis

A number of experiments using advanced techniques have demonstrated the influence of thermal vibration on allosteric processes in proteins outside the nervous system. I described these above (Hawkins and McLeish, 2006; Yonetani and Laberge, 2008; Teilum et al., 2009; Brust et al., 2013; Iakhiaev and Iakhiaev, 2013; Acbas et al., 2014; Müller-Werkmeister and Bredenbeck, 2014). These need to be repeated in the nervous system, preferably in simple organisms such as *Planaria* or *Drosophila*, using a series of memory and learning protocols. Further experiments in these preparations could be designed to investigate the postulated interactions between the three levels of neurocomputation.

## Conclusion

In the “bar-code” model of the synapse proposed in previous communications by Fuxe et al. (2014), the bar-code mechanism was formed by “lock-and-key,” allosteric interactions between the various proteins that make up the hetero-receptor complexes. We put forward here an addition to this hypothesis that molecular vibrations in proteins, particularly colored noise, may play a role in this process. Further experimental work is needed to tease out the contributions of each level of this hierarchy to the function of the neuron.

## Conflict of Interest Statement

The author declares that the research was conducted in the absence of any commercial or financial relationships that could be construed as a potential conflict of interest.
